# *Geobacillus* and *Bacillus* Spore Inactivation by Low Energy Electron Beam Technology: Resistance and Influencing Factors

**DOI:** 10.3389/fmicb.2018.02720

**Published:** 2018-11-23

**Authors:** Yifan Zhang, Ralf Moeller, Sophia Tran, Barbora Dubovcova, Georgios Akepsimaidis, Nicolas Meneses, David Drissner, Alexander Mathys

**Affiliations:** ^1^Sustainable Food Processing Laboratory, Institute of Food, Nutrition and Health, Department of Health Science and Technology, ETH Zurich, Zurich, Switzerland; ^2^Space Microbiology Research Group, Institute of Aerospace Medicine, Radiation Biology Division, German Aerospace Center, Cologne, Germany; ^3^Digital Technologies, Data Analytics and Services Business Unit, Bühler AG, Uzwil, Switzerland; ^4^Microbiology of Plant Foods, Agroscope, Waedenswil, Switzerland; ^5^Department of Life Sciences, Albstadt-Sigmaringen University, Sigmaringen, Germany

**Keywords:** bacterial spore, low energy electron beam, inactivation, influencing factors, surface decontamination, ionizing radiation, DNA damage

## Abstract

Low energy electron beam (LEEB) treatment is an emerging non-thermal technology that performs surface decontamination with a minimal influence on food quality. Bacterial spore resistance toward LEEB treatment and its influencing factors were investigated in this study. Spores from *Geobacillus* and *Bacillus* species were treated with a lab-scale LEEB at energy levels of 80 and 200 keV. The spore resistances were expressed as *D*-values (the radiation dose required for one log_10_ reduction at a given energy level) calculated from the linear regression of log_10_ reduction against absorbed dose of the sample. The results revealed that the spore inactivation efficiency by LEEB is comparable to that of other ionizing radiations and that the inactivation curves are mostly log_10_-linear at the investigated dose range (3.8 – 8.2 kGy at 80 keV; 6.0 – 9.8 kGy at 200 keV). The *D*-values obtained from the wildtype strains varied from 2.2 – 3.0 kGy at 80 keV, and from 2.2 – 3.1 kGy at 200 keV. *Bacillus subtilis* mutant spores lacking α/β-type small, acid-soluble spore proteins showed decreased *D*-values (1.3 kGy at 80 and 200 keV), indicating that spore DNA is one of the targets for LEEB spore inactivation. The results revealed that bacterial species, sporulation conditions and the treatment dose influence the spore LEEB inactivation. This finding indicates that for the application of this emerging technology, special attention should be paid to the choice of biological indicator, physiological state of the indicator and the processing settings. High spore inactivation efficiency supports the application of LEEB for the purpose of food surface decontamination. With its environmental, logistical, and economic advantages, LEEB can be a relevant technology for surface decontamination to deliver safe, minimally processed and additive-free food products.

## Introduction

Bacterial spores are the largest hurdle for perishable food preservation due to their extreme resistance to processing steps. Conventionally, food industries apply intensive decontamination processing steps (e.g., thermal preservation) alone or in combination with water activity and pH reduction to control bacterial spores. However, currently, consumers prefer to have fresh-looking and minimally processed food products that are safe and additive-free (Gould, 1996). Therefore, food industries and scientists have been continuously searching for novel non-thermal decontamination processes that can ensure microbiological safety as well as better preserve the freshness and nutritional value of the food products. Among the emerging decontamination technologies, low energy electron beam (LEEB) treatment has proved to be an effective bacterial inactivation with a minimal influence on food quality (Radomyski et al., 1994; Hayashi et al., 1997; De Lara et al., 2002; Arthur et al., 2005; Hertwig et al., 2018). LEEB treatment was introduced into the food industry as a sterilization method for packaging material in 2012 (Comet Group, 2012b) and recently entered into the spice and herb industries for decontamination purposes (International Irradiation Association [IIA], 2017).

Electron beam (EB) is a novel non-thermal sterilization technology, which is noninvasive and chemical-free. EB is a particle-based ionizing radiation, similar to photon-based X-rays and gamma rays, and inactivates bacteria by generating electrons. Generated electrons ionize, leading to breakage of target molecules through direct and indirect effects. Direct effects are damages caused by energy transfer of electrons to the target molecules, while indirect effects are damages induced by free radicals generated in the reaction of electrons with, e.g., water molecules (Tahergorabi et al., 2012; Lung et al., 2015). The exact target of the EB and its inactivation mechanism are still unclear, but it is suggested to be DNA, as seen in other ionizing irradiation technologies (Nicholson et al., 2000; Moeller et al., 2008, 2014).

Depending on the kinetic energy of the electrons, an EB can be distinguished as either a high energy electron beam (HEEB; >300 keV) or LEEB (≤300 keV) (ISO/ASTM 51818, 2009; Tallentire et al., 2010). The kinetic energy of the electrons and the density of the treated material determine the penetration depth. The higher the kinetic energy and the lower the density of the target are, the deeper the electrons can penetrate (Urgiles et al., 2007). The electrons with high kinetic energy can penetrate food products up to several cm, while the penetration depth of electrons with low kinetic energy is limited to a micrometer scale (Jaczynski and Park, 2003; Urgiles et al., 2007).

The emerging EB technology has some advantages over other ionizing irradiation technologies and conventional decontamination technologies, e.g., fumigation with chemicals and dry heat decontamination. Compared to radiation with gamma rays, EB technology does not use radioactive sources (Jaczynski and Park, 2003; Black and Jaczynski, 2006). While it takes gamma radiation minutes to hours to deliver a certain dose, EB can deliver the same dose in few seconds due to a higher dose rate (Silindir and Ozer, 2009; Fan et al., 2017). Moreover, since the electrons are generated electronically, EB can be tuned for the desired intensity and can be switched on or off instantly, which increases the control and flexibility of the application of this technology (Urgiles et al., 2007; ISO/ASTM 51818, 2009). Moreover, on top of these features, LEEB has shown some advantages in comparison to HEEB. LEEB technology deposits electron energy close to the surface where microorganisms are present, resulting in a higher efficiency for surface decontamination (Urgiles et al., 2007). Since the energy deposits are close to the surface, the product-process interactions occur mainly on the surface, resulting in less impact on food quality (Hayashi, 1998; De Lara et al., 2002; Kikuchi et al., 2003). For example, research suggested LEEB can achieve microbial decontamination without inducing much starch degradation (Hayashi et al., 1997) or influencing seed germination (Trinetta et al., 2011; Fan et al., 2017). Furthermore, with its compact size and a minimal need for shielding, LEEB technology is scalable to continuous processes and can be easily implemented in existing processing lines (Bugaev et al., 1994; Hayashi, 1998; Chalise et al., 2007).

Despite all the advantages mentioned above, LEEB is not yet widely applied in the food industry as a decontamination technology. One of the reasons for that might be the lack of consumer acceptance for irradiated foods (Schweiggert et al., 2007; Frewer et al., 2011; Junqueira-Goncalves et al., 2011; Finten et al., 2017). Part of the consumer resistance is due to lack of information and understanding of food irradiation or wrongly associating irradiated food with radioactive food (Maherani et al., 2016). In some cases, the consumers are concerned about the possible side effects of inductive radiation on irradiated food products and the use of radioactive energy (Sahasrabudhe, 1990; De Lara et al., 2002). However, consumer resistance toward this novel decontamination technology appears to be decreasing as consumers and food industries recognize that irradiation can be an effective alternative to chemical additives to preserve foods (Monk et al., 1995; DeRuiter and Dwyer, 2002; Sabharwal, 2013). Moreover, studies showed that consumer acceptance toward irradiated foods can be further improved by consumer education (DeRuiter and Dwyer, 2002; Nayga et al., 2005).

The other reason for its limited application in food industry so far could be that compared to other well-studied irradiation technologies such as gamma irradiation, only a limited amount of studies support the use of LEEB treatment as an efficient decontamination technology (De Lara et al., 2002; Tallentire et al., 2010; Fan et al., 2017). Most of the present LEEB studies are on specific foods, focusing on naturally presented microbial flora and often using different treatment setups (Hayashi, 1998; Rahman et al., 2006). Moreover, the inactivation efficiency was often reported as a reduction of microbial load instead of *D*-values (the radiation dose required for one log_10_ reduction at a given energy level), and often, the absorbed dose was not measured (Hayashi, 1998; Hayashi et al., 1998; Rahman et al., 2006). These reasons make it difficult to compare the inactivation efficiency of LEEB technology between different LEEB studies (Hayashi et al., 1997; Baba et al., 2004; Imamura et al., 2009), and to that of other conventional spore inactivation technologies, making it more challenging to validate this emerging technology.

Moreover, the efficiency and mechanism of LEEB on bacterial spore inactivation are rarely studied (Fiester et al., 2012). Therefore, more research must be performed for this technology to be utilized as a decontamination step. Bacterial spores are generally more resistant to irradiation treatment than vegetative cells, yeasts, and molds (van Gerwen et al., 1999; De Lara et al., 2002; Setlow, 2006, 2014). For example, Thayer and Boyd (1994) confirmed that *B. cereus* spores have a higher irradiation resistance than that of vegetative cells, and van Gerwen et al. (1999) showed that spores have significantly higher *D*-values than those of most vegetative bacteria, based on a total 539 *D*-values from 38 irradiation studies. A few vegetative bacteria have similar or higher irradiation resistance than that of bacterial spores (e.g., *Deinococcus radiodurans*), but those species are less relevant in the food industry and are not pathogenic.

In this study, we evaluated the spore inactivation efficiency of LEEB by determining the *D*-values for relevant *Geobacillus* and *Bacillus* species, calculated from the linear regression of log_10_ reduction against absorbed dose of the spore sample. The potential spore LEEB resistance influencing factors, including spore species, sporulation conditions and treatment kinetic energy, were also investigated. Additionally, the possible mechanism of spore inactivation by LEEB treatment was also investigated by using a mutant lacking α/β-type small, acid-soluble spore proteins (SASP), the major protection of spore DNA against damage (Setlow, 1995; Moeller et al., 2008, 2009).This study provided a comparison of LEEB spore inactivation efficiency with other published ionizing radiation decontamination data and gave additional information on the potential target of LEEB technology that induces spore inactivation. This will support the validation and application of the novel LEEB decontamination technology and help in the future delivery of safe, minimally processed and additive-free food products.

## Materials and Methods

### Bacterial Strains, Sporulation and Sample Preparation

In total, three bacterial species and one *B. subtilis* mutant were used in this study. This included *Geobacillus stearothermophilus* ATCC 7953, the biological indicator for the wet-heat sterilization process; *B. pumilus* DSM 492, the biological indicator for the irradiation sterilization process (Prince, 1976); *B. subtilis* wild-type PS 832, a model strain frequently used in spore research and its isogenic mutant *B. subtilis* PS 578 (termed as α^-^β^-^) that lacks the genes encoding the two major α/β-type small acid-soluble spore proteins (Nicholson and Setlow, 1990a; Fairhead et al., 1993).

*Bacillus subtilis* PS 832 and PS 578 were kindly provided by Dr. Peter Setlow and Dr. Barbara Setlow. *B. pumilus* DSM 492 was obtained from DSMZ (German Collection of Microorganisms and Cell Cultures GmbH). Spores of *G. stearothermophilus* ATCC 7953 were obtained as a commercial spore suspension from MesaLabs (France). Except for *G. stearothermophilus* ATCC 7953, all the others were sporulated at 30°C using modified Difco sporulation media (mDSM) agar plates, with nutrient broth pH 6.9 and without NaCl, from Sigma-Aldrich (Sigma-Aldrich, United States) instead of Difco, and the pH was adjusted to 7.2 (Nicholson and Setlow, 1990b). *B. subtilis* PS 832 was also sporulated at 37°C on mDSM and 2 × SG, a modified Schaeffer’s medium described previously (Leighton and Doi, 1971) to investigate the influence of the sporulation conditions on spore resistance toward LEEB treatment. Sporulation cultures were checked with a phase-contrast microscope (Leica, Germany) to estimate the percentages of dormant spores (phase-bright). Spores were harvested when the phase-bright spore percentage was >98%. Harvesting was performed by adding H_2_O (4°C) to the surface of the cultivation plates and suspending the overgrown colonies containing spores with sterile spreading sticks. The suspension was then transferred to a 50 ml centrifuge tube and washed with H_2_O (4°C) to remove the remaining vegetative cells, cell debris, and germinated spores. Spore stocks were stored in H_2_O at 4°C until usage.

A volume of 1 ml of spore suspension (around 10^9^ spores/ml; except for *G. stearothermophilus* ATCC 7953 which had an inoculation concentration of around 10^6^ spores/ml) was carefully dropped and spread on the upper surface of an autoclaved sterile microscope glass slide (Thermo Fisher Scientific, United States) that laid on a petri dish. The spore suspension stayed on the surface, and all slides were air-dried in a biosafety bench at room temperature. Afterward, the samples were stored and transported for treatment.

### Low Energy Electron Beam Treatment and Recovery

Samples were treated in the petri dish without a lid using a LEEB system EBLab-200 (Comet Group, Switzerland) at energy levels of 200 and 80 keV. The schematic of a LEEB lamp can be found elsewhere (Hertwig et al., 2018). Samples were either run through the machine without the electron source being turned on (0 kGy) or at nominal doses of 4, 5, 6, and 7 kGy. Due to the limited stability of the EB lamp at low electric current, treatment at lower doses was not performed. The distance between the emission window to samples was approximately 18 mm. Samples were treated under a N_2_ atmosphere (residual O_2_ < 210 ppm). All treatments were conducted at room temperature (approximately 23°C). Three independent samples were treated at each dose (results were calculated based on absorbed dose shown in Table 1) for all investigated spore strains.

**Table 1:** Absorbed dose of spore samples at 80 and 200 keV.

Nominal dose (kGy)	4	5	6	7
Absorbed dose at 80 keV (kGy)	3.8 ± 0.39	4.7 ± 0.64	6.6 ± 0.94	8.2 ± 0.86
Absorbed dose at 200 keV (kGy)	6.0 ± 0.28	7.0 ± 0.58	8.2 ± 0.62	9.8 ± 0.85


After samples were treated with LEEB, recovery was performed to enumerate cultivable survivors. Treated samples on microscope slides were put into 50 ml falcon tubes filled with 20 ml of 10 mM phosphate buffered saline (PBS, VWR International, United States) containing 0.04% Tween 80 (Sigma-Aldrich, United States). After vigorous shaking for 4 min, microscope slides were removed using flame-sterilized tweezers. The solution, which contained spores that washed off from the glass slides, was plated in triplicates onto tryptic soy agar (TSA, Sigma-Aldrich, United States) plates at appropriate dilutions. Plates were incubated at 37°C for *B. subtilis* and *B. pumilus* and 55°C for *G. stearothermophilus*. After incubation, the colony forming units (CFU) were counted. To derive the *D*-values reflecting the inactivation efficiency, spore survival fraction (N/N_0_) was plotted against the absorbed dose on a semi-logarithmic scale. Regression analysis was performed using Origin 9.1 (OriginLab Corporation, United States). The *D*-values were calculated from the slope of the linear regression of log_10_ reduction against absorbed dose according to equation (1). An average *D*-value (*n* = 3) was calculated for each strain. Differences between datasets were analyzed with Excel 2016 (Microsoft, United States), using two-tailed *t*-test with equal variance and a significance level of 0.05.

(1) $$D\;value=-\frac{1}{m}$$

Herein *m* is the slope of linear regression of log_10_ (N/N_0_) against absorbed dose.

### Dosimetry

The routine dosimeters used in this study were Risø B3-12 films (Risø High Dose Reference Laboratory, Denmark), which are 18 μm thick. The films were taped on microscope slides, placed in petri dishes and treated under the same conditions as the samples. The surface dose at each nominal dose used in this study was measured with three films placed at the same location as the samples with three replicate treatments. In total, nine films for each setting were irradiated and analyzed. Since electrons with low kinetic energy can be absorbed over a few micrometers, a dose gradient is created across the thickness of the Risø B3-12 dosimeter films that were used for dose measurement (Tallentire et al., 2010). The measured doses using Risø B3-12 dosimeter films were corrected to *D*_μ_, which is the absorbed dose in the first micrometer of the absorbing medium (Helt-Hansen et al., 2010). *D*_μ_-values were evaluated using Risøscan software with a calibration, which was obtained with the help of the Risø High Dose Reference Laboratory (HDRL, Denmark) for each applied energy level (80 and 200 keV) (Helt-Hansen et al., 2005). This calibration ensured that the reported doses from the low energy electron irradiations had measurement traceability to national standards (Helt-Hansen et al., 2010). The overall estimated uncertainty at *k* = 2 (a coverage factor *k* = 2 is close to a 95% confidence interval) of one dose measurement is around 10.6%. The overall uncertainty covers the uncertainty associated with calibration with alanine dosimeters, measurement of alanine dosimeter and *D*_μ_ determination.

In our situation, the spore samples were 1–2 μm thick, while the Risø B3-12 films, which is the thinnest standard dosimeter, are 18 μm thick. Therefore, the spore layer sits directly on the glass slide that served as a sample holder on the bottom, while the first micrometer of Risø B3-12 dosimeter are not directly in contact with the glass slide. Since a glass slide gives a stronger backscatter compared to the dosimeter, the spore samples were actually getting higher doses than *D*_μ_ that is measured by the dosimeter. Therefore, further simulations were done by the Risø High Dose Reference Laboratory (Denmark) concerning the effect of backscatter from different materials at different energy levels. Correction factors were obtained for the backscatter from the glass slide and from the dosimeter based on the simulation output. The measured *D*_μ_-values were further corrected to the absorbed dose of the spore samples based on the correction factors.

## Results

### Absorbed Dose of Spore Samples

Accurate dosimetry is essential for acquiring exact results, so the minimum and maximum measured doses were included when reporting EB inactivation experiments (Pillai and Shayanfar, 2017). Acquiring accurate surface doses for the low-energy range (e.g., 80 keV) was challenging due to the dose gradients within the treated dosimeter films. In this study, depth-dose distribution was established, and the surface dose *D*_μ_ was obtained using Risøscan software, calculated from measured apparent dose *D*_app_ (Helt-Hansen et al., 2010). The absorbed doses for our spore samples were corrected based on the surface dose *D*_μ_ and simulation output as described in Section “Dosimetry.” The absorbed doses of the spore samples at each nominal doses are shown in Table 1.

### Spore Inactivation

To investigate the spore inactivation efficiency by LEEB treatment and its influencing factors, spores obtained from different species and sporulation conditions were treated with LEEB at different kinetic energy levels, and their *D*-values were calculated and compared.

#### Kinetics

**FIGURE 1:**
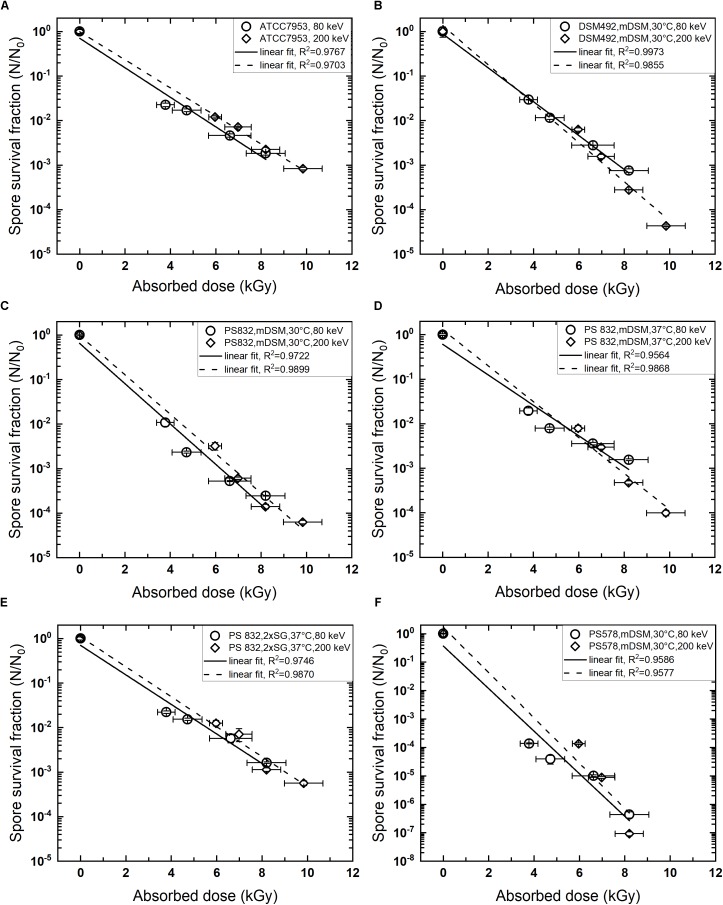
Kinetics of low energy electron beam spore inactivation. **(A)** *Geobacillus stearothermophilus* ATCC 7953, commercial spore suspension; **(B)** *Bacillus pumilus* DSM 492, sporulated on mDSM, 30°C; **(C)**: *B. subtilis* PS 832, sporulated on mDSM, 30°C; **(D)** *B. subtilis* PS 832, sporulated on mDSM, 37°C; **(E)** *B. subtilis* PS 832, sporulated on 2 × SG, 37°C; **(F)** *B. subtilis* PS 578 (α^-^β^-^ mutant), sporulated on mDSM, 30°C. Data are average ± standard deviation.

The regression analysis indicates a linear relationship between log_10_ reduction and absorbed dose used in this study for all species tested. The inactivation curves of different wildtype strains exhibited *R*^2^ > 0.95. The mutant PS 578 showed lower resistance to LEEB and when treated at 9.8 kGy (200 keV), the survivors were below detection limit. Therefore, only four data points were obtained under this condition and the *R*^2^ is higher than 0.95. All inactivation curves are shown in Figure 1.

#### Efficiency and Influencing Factors

The *D*-values obtained from this study for the wildtype strains varied from 2.2 – 3.0 kGy at 80 keV and 2.2 – 3.1 kGy at 200 keV. Different bacterial species showed diverse resistance toward LEEB treatment and the sporulation conditions, and the treatment energy levels showed influences on the spore inactivation efficiency. The spore resistance, expressed as *D*-values, is shown in Table 2. *B. subtilis* sporulated at 30°C on mDSM showed the lowest resistance toward LEEB treatment, with *D*-values of 2.2 kGy at 80 and 200 keV. Interestingly, *B. pumilus* DSM 492, which is suggested to be the biological indicator for irradiation sterilization, did not show higher resistance in most cases: it had lower resistance than that of *G. stearothermophilus* ATCC 7953, especially at the energy level of 200 keV, and it was less resistant than *B. subtilis* sporulated at 37°C on mDSM as well as on 2 × SG.

**Table 2:** *D*-values of spore inactivation under low energy electron beam treatment.

	*D*_80keV_ (kGy)	*D*_200keV_ (kGy)
*G. stearothermophilus* ATCC 7953	3.0 ± 0.03^a∗^	3.1 ± 0.05^e∗^
*B. pumilus* DSM 492, mDSM, 30°C	2.6 ± 0.00^b∗^	2.3 ± 0.02^f∗^
*B. subtilis* PS 832, mDSM, 30°C	2.2 ± 0.01^c^	2.2 ± 0.02^g^
*B. subtilis* PS 832, mDSM, 37°C	3.0 ± 0.07^a∗^	2.5 ± 0.03^h∗^
*B. subtilis* PS 832, 2 × SG, 37°C	2.9 ± 0.08^a^	2.9 ± 0.06^i^
*B. subtilis* PS 578, mDSM, 30°C	1.3 ± 0.07^d^	1.3 ± 0.02^j^


*G. stearothermophilus ATCC 7953 was obtained as a commercial spore suspension. Different lower case letters indicate statistically significant differences within the same column (*p* < 0.05). ^∗^indicate statistically significant different within the same row (*p* < 0.05).*

From Table 2, it was observed that *B. subtilis* PS 832 spores sporulated at the higher temperature had higher resistance to LEEB treatment at both energy levels. The *D*-values for spores that were sporulated at 37°C were approximately 0.3 kGy (200 keV) and 0.8 kGy (80 keV) higher than those sporulated at 30°C, even though both sets were sporulated on mDSM agar plates. At the same time, the nutrient richness of the sporulation media also showed influences on spore resistance to LEEB, as the spores sporulated on 2 × SG had higher resistance than that of the ones sporulated on mDSM when treated at 200 keV. The mutant *B. subtilis* PS 578 showed the smallest *D*-value, meaning it was much more sensitive to LEEB treatment than the other strains tested. The results obtained in this study suggest that in some cases there were no significant differences (*p* > 0.05) between LEEB treatment under 80 and 200 keV. Although for some samples, D_80*keV*_ is significantly different compared to D_200*keV*_, there was not a clear trend. For *G. stearothermophilus*, the *D*-values were slightly but statistically significantly (*p* = 0.01) lower at 80 keV compared to at 200 keV, with an average *D*-value of 3.0 kGy at 80 keV compared to 3.1 kGy at 200 keV. For *B. subtilis* PS 832 spores sporulated on mDSM, 37°C, the *D*-value at 200 keV was 0.5 kGy lower than at 80 keV, while for *B. pumilus* DSM 492, the *D*-value at 200 keV was 0.3 kGy lower than at 80 keV (in both cases, *p* < 0.01). These results should be taken with caution, as due to the lack of accurate dose measurement techniques at 1–2 μm layers, it is not possible to determinate accurately what the absorbed doses are. A more accurate dose assessment method might have an impact on the *D*-values.

## Discussion

### Spore Inactivation Kinetics and Efficiency

Linear spore inactivation kinetics ranging from 3.8 – 8.2 kGy at 80 keV and 6.0 – 9.8 kGy at 200 keV for different species were revealed in this study. Due to the limited stability of the EB lamp at low electric current, spore inactivation at lower doses was not investigated, and therefore, the possibility of a potential nonlinear log_10_ behavior at the range of lower doses cannot be excluded. Nevertheless, the linear inactivation found in the dose range used is in accordance with previous reports on LEEB inactivation of *B. pumilus* spores (Tallentire et al., 2010). The linear inactivation kinetics were also revealed in spore inactivation research of HEEB. For example, Fiester et al. (2012) reported a linear inactivation curve for *B. atrophaeus* treated at 5 MeV. However, nonlinear spore inactivation curves by HEEB were also reported in previous studies. For example, a nonlinear log_10_ inactivation curve with a shoulder formation was found for specific strains (Ito and Islam, 1994) and for *B. subtilis* spores that were sporulated in plate count agar (De Lara et al., 2002).

The *D*-values of the investigated wildtype strains in this study were in the range of 2.2 – 3.0 kGy at 80 keV and 2.2 – 3.1 kGy at 200 keV. These *D*-values were slightly higher compared to other LEEB studies (Urgiles et al., 2007; Tallentire et al., 2010). For example, the *D*-value for *B. pumilus* at 80 keV derived from this study was 2.6 kGy, while in the study by Tallentire et al. (2010) the *D*-value was 1.58 kGy, and by Urgiles et al. (2007), was 1.34 kGy. However, those differences in *D*-values could be due to different sample preparation methods, treatment conditions (e.g., at ambient atmosphere or N_2_ atmosphere) and recovery methods. For example, in the study of Urgiles et al. (2007), spores were inoculated on Al and Ti coupons, while in our study, spores were inoculated on glass slides. Moreover, the recovery solution and incubation temperature were also different.

The *D*-values obtained in this study are comparable to those reported for HEEB (Ito and Islam, 1994; De Lara et al., 2002). This is consistent to previous research (Tallentire et al., 2010; Gryczka et al., 2018). For example, Gryczka et al. (2018) described that under the treatment conditions applied, HEEB and LEEB treatments have a comparable efficiency in lowering the bacterial load of the food products investigated. Moreover, Tallentire et al. (2010) reported the response of *B. pumilus* spores found to be the same when treated with HEEB and LEEB. On the other hand, another study revealed that the *D*-values for *B. pumilus, B. subtilis*, and *B. megaterium* were slightly lower at LEEB treatment compared to HEEB, with *D*-values at 10 MeV were 2.12, 2.05, and 4.11 kGy, respectively, and at 100 keV were 1.34, 1.01, and 3.46 kGy (Urgiles et al., 2007).

In some studies, the inactivation efficiency of EB was similar to that of other ionization radiation types (Ohki et al., 1990; Ito and Islam, 1994; Van Calenberg et al., 1998; De Lara et al., 2002; Tallentire et al., 2010; Fiester et al., 2012). For example, Ohki et al. (1990) reported that the radiation sensitivity was almost equivalent when treated with gamma rays, X-rays, or EB, and the *D*-values obtained were 1.5 – 1.6 kGy for *B. pumilus* and 1.4 – 1.5 kGy for *B. subtilis*. The *D*-values obtained in our study are higher than those found by Ohki et al. (1990). They are also slightly higher than the estimated average *D*-values concluded by van Gerwen et al. (1999) for spores under irradiation treatment. In their study, the estimated *D*-value for spores treated with various irradiation processes was approximately 2.11 kGy, excluding some exceptions and specific conditions. However, as also stressed in their study, comparison of *D*-values from different projects should be acknowledged with care, since often the irradiation source is not the only influencing factor. In this study, spore species and sporulation conditions were also shown to affect spore inactivation efficiency of LEEB technology.

### Influencing Factors on Spore Inactivation Efficiency of LEEB

#### Spore Species

From our results, it can be seen that spores of *B. subtilis* and *B. pumilus* sporulated on mDSM, 30°C and *G. stearothermophilus* showed significant differences (*p* < 0.05) in their resistance to LEEB treatments (Table 2). This observation is consistent with other ionizing radiations, which revealed that spores of different species or even different strains showed different resistances (Anellis et al., 1972; Ito and Islam, 1994; Monk et al., 1995; van Gerwen et al., 1999). For example, previous research revealed that pathogenic *B. cereus* was more resistant than *B. subtilis* (De Lara et al., 2002). Therefore, for specific food applications, process parameters should be adjusted for relevant contamination microbiota to ensure sufficient inactivation.

Our study also revealed that the *D*-values of *G. stearothermophilus* ATCC 7953 were significantly higher (*p* < 0.01) than *B. pumilus* DSM 492, which is suggested to be the biological indicator for irradiation-based sterilization. The great resistance of *G. stearothermophilus* was also reported for other irradiation sources (Anellis and Koch, 1962; Briggs, 1966; Harnulv and Snygg, 1973; Radomyski et al., 1994; van Gerwen et al., 1999). For example, previous research revealed that *G. stearothermophilus* had greater gamma irradiation resistance than that of *Bacillus* species (e.g., *B. subtilis, B. megaterium*, and *B. cereus*) (Briggs, 1966; Harnulv and Snygg, 1973). Therefore, *G. stearothermophilus* might be more suitable as a biological indicator for LEEB treatment than *B. pumilus*. If using *G. stearothermophilus* ATCC 7953 spores as an indicator for radiation doses at 10 kGy, which is recommended as the maximum applicable dose to food (FAO/WHO Codex Alimentarius Commission, 2017), more than 3 log_10_ reduction can be achieved by using LEEB technology with the *D*-value obtained in this study. However, when applying to real food matrices, the spore inactivation efficiency of LEEB might be different, as the matrices might affect it. For example, the location of spores in food matrices, the water content, and the nutrient profile of the food matrices can influence the inactivation efficiency. On the other hand, the use of *B. megaterium* spores as a biological indicator should also be considered, since they displayed an even higher resistance toward LEEB than that of *G. stearothermophilus* in some studies (Ohki et al., 1990; Pillai et al., 2006).

#### Sporulation Conditions

It was found that the sporulation conditions also influence the inactivation of LEEB, with the higher sporulation temperature leading to increased spore resistance. This result could be because increased sporulation temperature lowers the spore core water content, leading to less indirect damage from hydroxyl radicals formed during irradiation (Beaman and Gerhardt, 1986; Moeller et al., 2008). Moreover, the sporulation media also played a role in spore resistance toward LEEB treatment, as the more nutrients (2 × SG) that were in the sporulation media, the more resistant the spores were toward the treatment. Sporulation media also showed an influence on spore inactivation by HEEB technology. For example, *B. subtilis* spores sporulated on plate count agar had a *D*-value of approximately 3.6 kGy, while when sporulated on nutrient agar, the *D*-value was approximately 1.5 kGy (De Lara et al., 2002). However, the nutrient richness of plate count agar and nutrient agar is comparable, so it might be the salt content in the media that influenced the spore resistance. Moreover, in their study, the inactivation kinetics for the spores that sporulated in these two different media were different. When sporulated on nutrient agar plates, the inactivation curve was linear, while with plate count agar, the inactivation curve appeared biphasic. This might also influenced their *D*-value evaluation. In our case, at the evaluated dose range (3.8 – 8.2 kGy at 80 keV and 6.0 – 9.8 kGy at 200 keV), although the resistance was different, the inactivation curves were all log_10_ linear. However, the possibility of a potential biphasic behavior out of the evaluated dose range cannot be excluded. Nevertheless, these influences mean that the physiological status of microorganisms should also be considered when evaluating the effectiveness of new decontamination technologies such as LEEB.

#### Kinetic Energy

Within the current limitations on dosimetry and the impact this might have on *D*-value calculations, our results showed that the kinetic energy level does not influence significantly the spore resistance for half of the investigated samples. No clear trend was found for the other half of samples, as 80 keV lead to a higher inactivation efficiency for *G. stearothermophilus*, while 200 keV lead to higher inactivation efficiencies for *B. pumilus* (mDSM, 30°C) and *B. subtilis* (mDSM, 37°C). Different theories concerning the influence of energy level of electrons on inactivation efficiency were proposed by previous studies (Nikjoo and Goodhead, 1991; Urgiles et al., 2007; Nikjoo and Lindborg, 2010; Krieger, 2012; Bellamy and Eckerman, 2013). A previous research stated that the inactivation is due to DNA bond-breakage, and these bond energies are approximately a few eV, which is considerably less than the energies of the irradiating electrons. Therefore, it should be the absorbed dose, instead of the energy of individual electrons, that determines the level of spore damage (Urgiles et al., 2007). Some other studies proposed that low-energy electrons lead to higher linear energy transfer, which is the amount of energy of an ionizing particle transferred to the biomolecule per unit distance, that in turn increases the relative biological effectiveness (Nikjoo and Goodhead, 1991; Nikjoo and Lindborg, 2010; Krieger, 2012; Bellamy and Eckerman, 2013).

#### Other Influencing Factors

Food matrices might also influence the spore inactivation efficiency of LEEB technology. In our study, 9 kGy at 80 keV and 200 keV could induce approximately 3 – 4 log_10_ reduction of different spore species on glass slides, while previous research revealed that approximately 9 kGy only induced around 2 log_10_ reduction of microorganisms present on soybeans (Kikuchi et al., 2003). Previous studies also revealed a great difference in spore resistance, depending on the kind of media on which spores were irradiated (Shamsuzzaman and Lucht, 1993). In addition, the presence of O_2_ during the treatment was suggested to influence the inactivation efficiency as well (Ito and Islam, 1994; Fiester et al., 2012). For example, the *D*-value of *B. megaterium* spores was increased from 1.8 to 5.1 kGy when they were vacuum-sealed under treatment (Ito and Islam, 1994). This could also be one of the reasons that the *D*-values in this study are slightly lower than some reported *D*-values from literature.

In summary, all these influencing factors should be taken into consideration when evaluating *D*-values. The *D*-values obtained from a model system might give a general information on the resistances of tested microorganisms toward LEEB treatment, but they might change their resistance significantly due to different sporulation and treatment conditions. Moreover, a more accurate dosimetry methodology should be developed for measurement of surface dose as this has a direct impact on the estimation of *D*-values. Therefore, it is very important to validate this novel LEEB technology for specific applications with the right dosimetry.

### DNA as One of the Targets for LEEB Spore Inactivation

From the results, we can observe the mutant that lacking SASP, which is the major protection of spore DNA, showed significant lower resistance than that of their isogenic wildtype (*p* < 0.01). This observation indicates that DNA is one of the targets of LEEB spore inactivation, which is similar to the findings using HEEB treatment (Fiester et al., 2012). In Fiester et al. (2012), they also found structural damage of the spore inner membrane and coat, in addition to DNA fragmentation, when *B. atrophaeus* spores were treated with HEEB at 5 MeV, and all of these changes correlated with the applied dose. This finding indicates that DNA is not the only target for HEEB spore inactivation, and whether this is also the case for LEEB requires additional investigation. Moreover, other studies revealed that the mutant lacking SASP also showed increased sensitivity to other ionizing irradiations (e.g., X-ray and high-energy charged iron ions); however, it seems that the lack of SASP does not affect spores’ resistance to gamma radiation (Nicholson et al., 2000; Setlow, 2006, 2007; Moeller et al., 2008, 2014).

## Conclusion and Recommendation

This study quantified the spore inactivation efficiency of LEEB treatment by evaluating the *D*-values for relevant species. The inactivation efficiency of LEEB technology is in a comparable range to that of the other ionizing irradiation technologies. However, the comparison between different studies should be taken with care, as disclosed in this study that several factors, including spore species and sporulation media can influence the spore inactivation efficiency of LEEB. This result indicates that for the application of this emerging technology, special attention should be paid to the choice of biological indicator, physiological state of the indicator, dosimetry, and the processing settings. Moreover, the highly efficient surface decontamination of LEEB treatment comes with a low penetration depth, which means the location of the food contaminants should also be carefully considered. The *B. subtilis* mutant experiments also revealed that one of the spore inactivation mechanisms of LEEB technology is to cause DNA damage. Future research on investigation of the nature and level of DNA damages and other damages induced by LEEB, as well as how can spores overcome the damages should be conducted to understand the inactivation mechanism of LEEB.

In general, high spore inactivation efficiency supports the application of LEEB technology for the purpose of food surface decontamination (e.g., for spices or sprouting seeds). Due to the environmental, logistical, and economic advantages of LEEB treatment, it would be a more practical alternative to other irradiation technologies for surface decontamination and could help deliver safe, minimally processed and additive-free food products.

## Author Contributions

YZ and ST performed the experiments with the support of all authors. GA contributed on the absorbed dose evaluation. All authors discussed the results and implications and commented on the manuscript at all stages.

## Conflict of Interest Statement

The authors declare that the research was conducted with the support of ETH Zurich Foundation, Bühler AG and DLR grant DLR-FuE-Projekt ISS LIFE, Programm RF-FuW, Teilprogramm 475.

## References

[B1] Anellis A. Berkowitz D. Swantak W. Strojan C. (1972). Radiation sterilization of prototype military foods: low-temperature irradiation of codfish cake, corned beef, and pork sausage. *Appl. Microbiol.* 24 453–462. 4562483 10.1128/am.24.3.453-462.1972 PMC376541

[B2] Anellis A. Koch R. B. (1962). Comparative resistance of strains of *Clostridium botulinum* to gamma rays. *Appl. Microbiol.* 10 326–330. 13861547 10.1128/am.10.4.326-330.1962 PMC1057868

[B3] Arthur T. M. Wheeler T. L. Shackelford S. D. Bosilevac J. M. Nou X. W. Koohmaraie M. (2005). Effects of low-dose, low-penetration electron beam irradiation of chilled beef carcass surface cuts on *Escherichia coli* O157: H7 and meat quality. *J. Food Protoc.* 68 666–672. 10.4315/0362-028x-68.4.666 15830654

[B4] Baba T. Kaneko H. Taniguchi S. (2004). Soft electron processor for surface sterilization of food material. *Radiat. Phys. Chem.* 71 209–211. 10.1016/j.radphyschem.2004.03.079

[B5] Beaman T. C. Gerhardt P. (1986). Heat resistance of bacterial spores correlated with protoplast dehydration, mineralization, and thermal adaptation. *Appl. Environ. Microbiol.* 52 1242–1246. 3098170 10.1128/aem.52.6.1242-1246.1986 PMC239215

[B6] Bellamy M. Eckerman K. (2013). *Relative Biological Effectiveness of Low-Energy Electrons and Photons.* Washington, DC: U. S. Environmental Protection Agency.

[B7] Black J. L. Jaczynski J. (2006). Temperature effect on inactivation kinetics of *Escherichia coli* O157 : H7 by electron beam in ground beef, chicken breast meat, and trout fillets. *J. Food Sci.* 71 M221–M227. 10.1111/j.1750-3841.2006.00105.x

[B8] Briggs A. (1966). The resistances of spores of the genus *Bacillus* to phenol, heat and radiation. *J. Appl. Bacteriol.* 29 490–504. 10.1111/j.1365-2672.1966.tb03500.x 5980916

[B9] Bugaev S. P. Korovin S. D. Kutenkov D. P. Landi V. F. Mesyats G. A. Sakharov E. S. (1994). “Surface sterilization using low-energy nanosecond pulsed electron beams,” in *Proceedings of the 10th International Conference on High-Power Particle Beams*, (San Diego, CA: IET), 817–820.

[B10] Chalise P. R. Hotta E. Matak K. E. Jaczynski J. (2007). Inactivation kinetics of *Escherichia coli* by pulsed electron beam. *J. Food Sci.* 72 M280–M285. 10.1111/j.1750-3841.2007.00451.x 17995653

[B11] Comet Group (2012a). *e-Beam Technology.* Flamatt: Brochure.

[B12] Comet Group (2012b). *Tetra Pak Unveils COMET ’s Innovative e-Beam Technology at Anuga 2012.* Available at: http://www.comet-group.com/news/2012/03/tetra-pak-unveils-comet-s-innovative-e-beam-technology-at-anuga-2012 [accessed September 11, 2017]

[B13] De Lara J. Fernández P. S. Periago P. M. Palop A. (2002). Irradiation of spores of *Bacillus cereus* and *Bacillus subtilis* with electron beams. *Innov. Food Sci. Emerg. Technol.* 3 379–384. 10.1016/S1466-8564(02)00053-X

[B14] DeRuiter F. E. Dwyer J. (2002). Consumer acceptance of irradiated foods: dawn of a new era? *Food Service Technol.* 2 47–58. 10.1046/j.1471-5740.2002.00031.x

[B15] Fairhead H. Setlow B. Setlow P. (1993). Prevention of DNA damage in spores and in vitro by small, acid-soluble proteins from *Bacillus* species. *J. Bacteriol.* 175 1367–1374. 10.1128/jb.175.5.1367-1374.1993 8444799 PMC193223

[B16] Fan X. T. Sokorai K. Weidauer A. Gotzmann G. Rogner F. H. Koch E. (2017). Comparison of gamma and electron beam irradiation in reducing populations of *E-coil* artificially inoculated on mung bean, clover and fenugreek seeds, and affecting germination and growth of seeds. *Radiat. Phys. Chem.* 130 306–315. 10.1016/j.radphyschem.2016.09.015

[B17] FAO/WHO Codex Alimentarius Commission. (2017). *Codex Alimentarius, in: General Standard for Irradiated Food.* Rome: Food and Agriculture Organization of the United Nations.

[B18] Fiester S. E. Helfinstine S. L. Redfearn J. C. Uribe R. M. Woolverton C. J. (2012). Electron beam irradiation dose dependently damages the *Bacillus* spore coat and spore membrane. *Int. J. Food Microbiol* 2012:9. 10.1155/2012/579593 22319535 PMC3272845

[B19] Finten G. Garrido J. I. Agüero M. V. Jagus R. J. (2017). Irradiated ready-to-eat spinach leaves: How information influences awareness towards irradiation treatment and consumer’s purchase intention. *Radiat. Phys. Chem.* 130 247–251. 10.1016/j.radphyschem.2016.09.004

[B20] Frewer L. J. Bergmann K. Brennan M. Lion R. Meertens R. Rowe G. (2011). Consumer response to novel agri-food technologies: Implications for predicting consumer acceptance of emerging food technologies. *Trends Food Sci. Technol.* 22 442–456. 10.1016/j.tifs.2011.05.005

[B21] Gould G. W. (1996). Industry perspectives on the use of natural antimicrobials and inhibitors for food applications. *J. Food Protoc.* 59 82–86. 10.4315/0362-028X-59.13.82 28384019

[B22] Gryczka U. Migdal W. Bulka S. (2018). The effectiveness of the microbiological radiation decontamination process of agricultural products with the use of low energy electron beam. *Radiat. Phys. Chem.* 143 59–62. 10.1016/j.radphyschem.2017.09.020

[B23] Harnulv B. G. Snygg B. G. (1973). Radiation resistance of spores of *Bacillus subtilis* and *B. stearothermophilus* at various water activities. *J. Appl. Bacteriol.* 36 677–682. 10.1111/j.1365-2672.1973.tb04152.x 4207058

[B24] Hayashi T. (1998). Decontamination of dry food ingredients with “soft-electrons” (low-energy electrons). *Jarq-Jpn Agr Res Q* 32 293–299. 10.3136/fsti9596t9798.4.114

[B25] Hayashi T. Okadome H. Toyoshima H. Todoriki S. Ohtsubo K. (1998). Rheological properties and lipid oxidation of rice decontaminated with low-energy electrons. *J. Food Prot.* 61 73–77. 10.4315/0362-028x-61.1.73 9708256

[B26] Hayashi T. Takahashi Y. Todoriki S. (1997). Low-energy electron effects on the sterility and viscosity of grains. *J. Food Sci.* 62 858–860. 10.1111/j.1365-2621.1997.tb15472.x

[B27] Helt-Hansen J. Miller A. Sharpe P. (2005). Dose response of thin-film dosimeters irradiated with 80-120 keV electrons. *Radiat Phys. Chem.* 74 341–353. 10.1016/j.radphyschem.2005.06.004

[B28] Helt-Hansen J. Miller A. Sharpe P. Laurell B. Weiss D. Pageau G. (2010). Dμ -A new concept in industrial low-energy electron dosimetry. *Radiat Phys Chem* 79 66–74. 10.1016/j.radphyschem.2009.09.002

[B29] Hertwig C. Meneses N. Mathys A. (2018). Cold atmospheric pressure plasma and low energy electron beam as alternative nonthermal decontamination technologies for dry food surfaces: a review. *Trends Food Sci. Technol.* 77 131–142. 10.1016/j.tifs.2018.05.011

[B30] Imamura T. Todoriki S. Miyanoshita A. Horigane A. K. Yoshida M. Hayashi T. (2009). Efficacy of soft-electron (low-energy electron) treatment for disinfestation of brown rice containing different ages of the maize weevil, *Sitophilus zeamais* Motschulsky. *Radiat Phys. Chem.* 78 627–630. 10.1016/j.radphyschem.2009.03.058

[B31] International Irradiation Association [IIA] (2017). *Decontamination of Dry Food Ingredients by Low Energy Electron Beam: a World First.* Available at: http://iiaglobal.com/news/decontamination-dry-food-ingredients-low-energy-electron-beam-world-first/ [accessed September 29, 2017]

[B32] ISO/ASTM 51818. (2009). *Practice for Dosimetry in an Electron Beam Facility for Radiation Processing at Energies between* 80 and 300 keV. West Conshohocken, PA: ASTM International.

[B33] Ito H. Islam M. S. (1994). Effect of dose rate on inactivation of microorganisms in spices by electron-beams and gamma-rays irradiation. *Radiat. Phys. Chem.* 43 545–550. 10.1016/0969-806X(94)90165-1

[B34] Jaczynski J. Park J. W. (2003). Microbial inactivation and electron penetration in surimi seafood during electron beam processing. *J. Food Sci.* 68 1788–1792. 10.1111/j.1365-2621.2003.tb12330.x

[B35] Junqueira-Goncalves M. P. Galotto M. J. Valenzuela X. Dinten C. M. Aguirre P. Miltz J. (2011). Perception and view of consumers on food irradiation and the Radura symbol. *Radiat. Phys. Chem.* 80 119–122. 10.1016/j.radphyschem.2010.08.001

[B36] Kikuchi O. K. Todoriki S. Saito M. Hayashi T. (2003). Efficacy of soft-electron (low-energy electron beam) for soybean decontamination in comparison with gamma-rays. *J. Food Sci.* 68 649–652. 10.1111/j.1365-2621.2003.tb05725.x

[B37] Krieger H. (2012). *Grundlagen der Strahlungsphysik und des Strahlenschutzes.* Wiesbaden, Germany: Springer Spektrum 10.1007/978-3-8348-2238-3

[B38] Leighton T. J. Doi R. H. (1971). The stability of messenger ribonucleic acid during sporulation in *Bacillus subtilis*. *J. Biol. Chem.* 246 3189–3195. 4995746

[B39] Lung H. M. Cheng Y. C. Chang Y. H. Huang H. W. Yang B. B. Wang C. Y. (2015). Microbial decontamination of food by electron beam irradiation. *Trends Food Sci. Technol.* 44 66–78. 10.1016/j.tifs.2015.03.005

[B40] Maherani B. Hossain F. Criado P. Ben-Fadhel Y. Salmieri S. Lacroix M. (2016). World market development and consumer acceptance of irradiation technology. *Foods* 5:79. 10.3390/foods5040079 28231173 PMC5302430

[B41] Moeller R. Raguse M. Reitz G. Okayasu R. Li Z. Klein S. (2014). Resistance of *Bacillus subtilis* spore DNA to lethal ionizing radiation damage relies primarily on spore core components and DNA repair, with minor effects of oxygen radical detoxification. *Appl. Environ. Microbiol.* 80 104–109. 10.1128/AEM.03136-13 24123749 PMC3911009

[B42] Moeller R. Setlow P. Horneck G. Berger T. Reitz G. Rettberg P. (2008). Roles of the major, small, acid-soluble spore proteins and spore-specific and universal DNA repair mechanisms in resistance of *Bacillus subtilis* spores to ionizing radiation from X rays and high-energy charged-particle bombardment. *J. Bacteriol.* 190 1134–1140. 10.1128/JB.01644-07 18055591 PMC2223577

[B43] Moeller R. Setlow P. Reitz G. Nicholson W. L. (2009). Roles of small, acid-soluble spore proteins and core water content in survival of *Bacillus subtilis* spores exposed to environmental solar UV radiation. *Appl. Environ. Microbiol.* 75 5202–5208. 10.1128/AEM.00789-09 19542328 PMC2725452

[B44] Monk J. D. Beuchat L. R. Doyle M. P. (1995). Irradiation inactivation of food-borne microorganisms. *J. Food Protoc.* 58 197–208. 10.4315/0362-028X-58.2.197 31121676

[B45] Nayga RM Jr Aiew W. Nichols J. P. (2005). Information effects on consumers’ willingness to purchase irradiated food products. *Rev. Agric. Econ.* 27 37–48. 10.1111/j.1467-9353.2004.00206.x

[B46] Nicholson W. L. Munakata N. Horneck G. Melosh H. J. Setlow P. (2000). Resistance of *Bacillus* endospores to extreme terrestrial and extraterrestrial environments. *Microbiol. Mol. Biol. Rev.* 64 548–572. 10.1128/MMBR.64.3.548-572.2000 10974126 PMC99004

[B47] Nicholson W. L. Setlow P. (1990a). Dramatic increase in negative superhelicity of plasmid DNA in the forespore compartment of sporulating cells of *Bacillus subtilis*. *J. Bacteriol.* 172 7–14. 10.1128/jb.172.1.7-14.1990 2104613 PMC208394

[B48] Nicholson W. L. Setlow P. (1990b). *Molecular Biological Methods for Bacillus.* New York, NY: John Wiley, 391–450.

[B49] Nikjoo H. Goodhead D. T. (1991). Track structure analysis illustrating the prominent role of low-energy electrons in radiobiological effects of low-LET radiations. *Phys. Med. Biol.* 36:229. 10.1088/0031-9155/36/2/007 2008448

[B50] Nikjoo H. Lindborg L. (2010). RBE of low energy electrons and photons. *Phys Med Bio* 55:R65. 10.1088/0031-9155/55/10/R01 20427859

[B51] Ohki Y. Ito H. Watanabe Y. Sunaga H. Ishigaki I. (1990). Comparative sensitivity of endospores from some *Bacillus* species to gamma-rays, X-rays and electron beams for sterilization. *Shokuhin Shosha* 25 71–74.

[B52] Pillai S. D. Shayanfar S. (2017). Electron beam processing of fresh produce – A critical review. *Radiat. Phys. Chem.* 143 85–88. 10.1016/j.radphyschem.2017.09.008

[B53] Pillai S. D. Venkateswaran K. Cepeda M. Soni K. Mittasch S. Maxim J. (2006). “Electron beam (10 MeV) irradiation to decontaminate spacecraft components for planetary protection,” in *Proceedings of the Aerospace Conference, 2006 IEEE*, (Big Sky, MT: IEEE). 10.1109/AERO.2006.1655743

[B54] Prince H. N. (1976). Stability of *Bacillus pumilus* spore strips used for monitoring radiation sterilization. *Appl. Environ. Microbiol.* 31 999–1000. 779652 10.1128/aem.31.6.999-1000.1976 PMC169869

[B55] Radomyski T. Murano E. A. Olson D. G. Murano P. S. (1994). Elimination of pathogens of significance in food by low-dose Irradiation: a review. *J. Food Protoc.* 57 73–86. 10.4315/0362-028X-57.1.73 31113022

[B56] Rahman M. S. Ghomi H. Chalise P. R. Hayashi Y. Watanabe M. Okino A. (2006). Inactivation of cells and spores of *Bacillus subtilis* using low energy pulsed electron beam. *Jpn. J. Appl. Phys.* 2 45 L881–L883. 10.1143/Jjap.45.L881

[B57] Sabharwal S. (2013). “Electron beam irradiation applications,” in *Proceedings of the PAC2013 Pasadena, CA: USA*, (Vienna: International Atomic Energy Agency), 745–748.

[B58] Sahasrabudhe M. R. (1990). Food irradiation: current status, concerns, limitations and future prospects. *J. Can. Diet. Assoc.* 51 329–334.

[B59] Schweiggert U. Carle R. Schieber A. (2007). Conventional and alternative processes for spice production - a review. *Trends Food Sci. Technol.* 18 260–268. 10.1016/j.tifs.2007.01.005

[B60] Setlow P. (2014). Spore resistance properties. *Microbiol. Spectr.* 2 201–215. 10.1128/microbiolspec.TBS-0003-2012 26104355

[B61] Setlow P. (1995). Mechanisms for the prevention of damage to DNA in spores of *Bacillus* species. *Annu. Rev. Microbiol.* 49 29–54. 10.1146/annurev.mi.49.100195.000333 8561462

[B62] Setlow P. (2006). Spores of *Bacillus subtilis*: their resistance to and killing by radiation, heat and chemicals. *J. Appl. Microbiol.* 101 514–525. 10.1111/j.1365-2672.2005.02736.x 16907802

[B63] Setlow P. (2007). I will survive: DNA protection in bacterial spores. *Trends Microbiol.* 15 172–180. 10.1016/j.tim.2007.02.004 17336071

[B64] Shamsuzzaman K. Lucht L. (1993). Resistance of *Clostridium sporogenes* spores to radiation and heat in various nonaqueous suspension media. *J. Food Protoc.* 56 10–12. 10.4315/0362-028x-56.1.10 31084047

[B65] Silindir M. Ozer A. Y. (2009). Sterilization methods and the comparison of e-beam sterilization with gamma radiation sterilization. *FABAD J. Pharm. Sci.* 34 43–53. 10.1002/mame.201600133 28280451 PMC5340269

[B66] Tahergorabi R. Matak K. E. Jaczynski J. (2012). Application of electron beam to inactivate *Salmonella* in food: recent developments. *Food Res Int* 45 685–694. 10.1016/j.foodres.2011.02.003

[B67] Tallentire A. Miller A. Helt-Hansen J. (2010). A comparison of the microbicidal effectiveness of gamma rays and high and low energy electron radiations. *Radiat. Phys. Chem.* 79 701–704. 10.1016/j.radphyschem.2010.01.010

[B68] Thayer D. W. Boyd G. (1994). Control of enterotoxic *Bacillus cereus* on poultry or red meats and in beef gravy by gamma irradiation. *J. Food Protoc.* 57 758–764. 10.4315/0362-028x-57.9.758 31121797

[B69] Trinetta V. Vaidya N. Linton R. Morgan M. (2011). A comparative study on the effectiveness of chlorine dioxide gas, ozone gas and e-beam irradiation treatments for inactivation of pathogens inoculated onto tomato, cantaloupe and lettuce seeds. *Int. J. Food Microbiol.* 146 203–206. 10.1016/j.ijfoodmicro.2011.02.014 21411164

[B70] Urgiles E. Wilcox J. Montes O. Osman S. Venkateswaran K. Cepeda M. (2007). “Electron beam irradiation for microbial reduction on spacecraft components,” in *Proceedings of the 2007 IEEE Aerospace Conference*, (Big Sky, MT: IEEE), 1–15. 10.1109/AERO.2007.352739

[B71] Van Calenberg S. Vanhaelewyn G. Van Cleemput O. Callens F. Mondelaers W. Huyghebaert A. (1998). Comparison of the effect of X-ray and electron beam irradiation on some selected spices. *Food Sci. Technol.* 31 252–258. 10.1006/fstl.1997.0352

[B72] van Gerwen S. J. C. Rombouts F. M. Riet K. V. T. Zwietering M. H. (1999). A data analysis of the irradiation parameter D10 for bacteria and spores under various conditions. *J. Food Protoc.* 62 1024–1032. 10.4315/0362-028X-62.9.1024 10492477

